# Supplementary material for: Identification of Prescribing Patterns in Hemodialysis Outpatients Taking Multiple Medications
Source: Pharmacy (Basel). 2023 Feb 23;11(2):43. doi: 10.3390/pharmacy11020043 (PMC10037568; doi:10.3390/pharmacy11020043)
Supplement: Supplementary file 1 [file pharmacy-11-00043-s001.zip › pharmacy-2214444-supplementary.pdf]

Supplementary table S1.

Univariate and multivariate logistic regression to assess the association between multidrug administration and specific comorbidities

Number of patients receiving polypharmacy (≥9 drugs/day)

	Univariate			Multivariate		
	OR	95% Confidence Interval		OR	95% Confidence Interval	
		Lower	Upper		Lower	Upper
Diabetes mellitus	2.82	1.23	6.44	2.88	1.21	6.87
Cardiovascular disease	2.01	0.87	4.68	2.09	0.86	5.06
Cerebrovascular disease	1.40	0.46	4.27	1.87	0.54	6.41
Peripheral artery disease	2.61	0.65	10.48	2.11	0.48	9.15
Liver disease	0.87	0.28	2.69	1.03	0.31	3.41
